# A latent variable approach to account for correlated inputs in global sensitivity analysis

**DOI:** 10.1007/s10928-021-09764-x

**Published:** 2021-05-25

**Authors:** Nicola Melillo, Adam S. Darwich

**Affiliations:** 1 Centre for Applied Pharmacokinetic Research, Division of Pharmacy & Optometry, School of Health Sciences, The University of Manchester, Manchester, UK; 2 Division of Health Informatics and Logistics, Department of Biomedical Engineering and Health Systems, KTH Royal Institute of Technology, Stockholm, Sweden

**Keywords:** Latent variable, Correlated factors, Global sensitivity analysis, Physiologically based pharmacokinetic models, Model-informed drug discovery and development

## Abstract

In drug development decision-making is often supported through model-based methods, such as physiologically-based pharmacokinetics (PBPK). Global sensitivity analysis (GSA) is gaining use for quality assessment of model-informed inference. However, the inclusion and interpretation of correlated factors in GSA has proven an issue. Here we developed and evaluated a latent variable approach for dealing with correlated factors in GSA. An approach was developed that describes the correlation between two model inputs through the causal relationship of three independent factors: the latent variable and the unique variances of the two correlated parameters. The latent variable approach was applied to a set of algebraic models and a case from PBPK. Then, this method was compared to Sobol’s GSA assuming no correlations, Sobol’s GSA with groups and the Kucherenko approach. For the latent variable approach, GSA was performed with Sobol’s method. By using the latent variable approach, it is possible to devise a unique and easy interpretation of the sensitivity indices while maintaining the correlation between the factors. Compared methods either consider the parameters independent, group the dependent variables into one unique factor or present difficulties in the interpretation of the sensitivity indices. In situations where GSA is called upon to support model-informed decision-making, the latent variable approach offers a practical method, in terms of ease of implementation and interpretability, for applying GSA to models with correlated inputs that does not violate the independence assumption. Prerequisites and limitations of the approach are discussed.

**Supplementary Information:**

The online version supplementary material available at 10.1007/s10928-021-09764-x.

## Introduction

In pharmaceutical research and development (R&D) decision-making is often supported by modelling and simulation (M&S), referred to as model-informed drug discovery and development (MID3) [56]. Physiologically-based pharmacokinetic (PBPK) M&S provides a framework for mechanistic predictions of in vivo drug exposure. PBPK M&S has replaced/supplemented clinical trials and informed labelling for numerous drugs, most notably for dosage recommendations following metabolic drug–drug interactions [22, 59, 59].

Uncertainty and variability are prominent in biological data. In this context, uncertainty mainly relates to inter- and intra-experimental variability and errors, as well as translation of parameters. Variability mainly relates to interindividual variability in physiology, interoccasion variability and more. Correlations between input parameters are often implemented in PBPK models to account for physiological constraints, otherwise causing implausible combinations of parameters [44, 53]. For example, organ weights are constrained by body weight. With the emergence of novel ‘omics techniques, the correlation of proteins is also of increasing interest [12, 37].

Sensitivity analysis (SA) and global SA (GSA) are essential instruments for the quality assessment of model-based inference [43] and their use has gained interest from pharmaceutical industry and academia in recent years [10, 26, 34, 36–38, 58, 60]. Moreover, both the United States Food and Drug Administration (FDA) and European Medicines Agency (EMA) have highlighted the importance of SA and GSA as best practice in PBPK to inform model development and refinement [6, 7]. GSA is key for elucidating the relationship between the uncertainty and variability in model inputs and variation in a given model output. For example, GSA can be a valuable method for testing if the model behaves as expected and, if not, it can provide useful information that helps in identifying the reasons and possible errors in the model assumptions or implementation. Moreover, GSA can help identify what parameters may need to be more precisely characterised to allow reliable model predictions [36, 38, 50]. Therefore, by extension, the method is relevant for decision-making informed by modelling in drug development and clinical practice [17, 36, 37, 47, 58].

In this work, we focused on the variance-based GSA (also referred to as Sobol’s method) [36, 38]. This choice was made as variance-based GSA is able to handle nonlinear and nonmonotonic relationships between the input factors and the model outputs [49–51]. Moreover, with this method it is possible to quantify the effect of each factor taken singularly and the extent of its interaction effects. As we have reported in our previous work, understanding the extent of the interaction effects can be particularly important for an informed use of PBPK models during drug development [38].

The classical variance-based GSA works under the assumption that model inputs (commonly referred to as model parameters in pharmacometrics) are independent [49–51]. Under this assumption, the variance decomposition is unique [51] and reflects the structure of the model itself [40]. In this context, the variance-based sensitivity indices have a clear interpretation [21, 49]. However, it is not uncommon that PBPK models violate the independence assumption [26, 37, 53]. In practice this may lead to correlations being ignored in the analysis, or the use of one of several proposed methods for GSA that deal with dependent inputs. Perhaps, the most simple and elegant way of treating dependent inputs in GSA is by grouping the correlated factors and then performing a GSA with the independent groups. The intrinsic limitation of this approach is that it is not possible to distinguish the contribution of the single variables within each group.

In the literature, several methods have been developed to deal with dependent inputs while retaining the information, or sensitivity indices, of each individual factor. These methods can be classified into two categories: parametric and non-parametric methods [11, 31]. The parametric methods, also called model-based methods, (e.g., [9, 25, 57]) assume an a priori model for the input-output relation. Instead, the non-parametric approaches do not assume any specific shape for this relation and thus, they are referred to as model-free or non model-based methods [11, 31]. These approaches were considered to be more suitable for computer-based modelling [11]. Generally, the non-parametric methods employ a transformation technique for dealing with correlated factor distributions [11]. For example, Kucherenko et al. [24] used copula transformations to generalise the first order and total Sobol indices for the case of dependent input factors. Mara et al. [31] proposed the use of the Rosenblatt transformation, and Tarantola and Mara [52] used both the Rosenblatt and Nataf transformation within the context of variance-based GSA. Moreover, other methods such as the variogram analysis of response surfaces (VARS) and the Shapely effects have been extended for the case of correlated input factors [11, 21].

The copula-based method, developed by Kucherenko et al. [24], has recently been proposed for PBPK models [26]. However, how to interpret variance-based GSA results in presence of dependent variables is not straightforward and still debated among GSA practitioners. In presence of correlation between the input factors, the correspondence between the variance-based indices and model structure is lost and the variance decomposition can no longer provide a description of the model structure [3, 40, 42]. This was illustrated by Oakley and O’Hagan in 2004 with the use of a simple example [40]. In this context, Pianosi et al. reported that *counterintuitive results may be obtained* [42]. Iooss and Lemaître reported that *SA for dependent inputs has also been discussed by several authors [...], but this issue remains misunderstood* [20]. Moreover, Iooss and Prieur reported that *The so-called Sobol’ indices [...], present a difficult interpretation in the presence of statistical dependence between inputs* [21]. Finally, a recent position paper Razavi et al. reported that *The field of SA in terms of methods to handle input constraints and correlation structures is still embryonic* [43].

Several dedicated software platforms exist for PBPK M&S [23], providing accessible tools for non-expert users. As GSA gains use in the community (such as through software implementation) the issue of interpretability becomes increasingly relevant.

Here we propose a latent variable approach for treating correlated input parameters in variance-based GSA. The method expresses the correlation between two parameters as causal relationships between uncorrelated variables. This is done in order to allow the use of classical variance-based GSA and avoids the usage of methods whose interpretation is still a matter of debate. Latent variable models and sub-varieties of them, such as factor analysis, path analysis and structural equation modelling, are widely used in social sciences [28]. In latent variable models, the correlation between more than one observed measure (or model parameter) is described by one, or more, unobserved (latent) variable(s). Parameters are correlated as they share a common cause [4]. Here we focus on the case of two linearly correlated random variables whose correlation is explained by one latent variable. With this approach, instead of two correlated factors, three independent factors (the latent variable and the two independent variances of the correlated parameters) are considered in the GSA.

The approach is then applied to a set of algebraic models and a whole-body PBPK model for the drug midazolam (MDZ). MDZ is a sedative primarily metabolised by Cythochrome P450 (CYP) 3A4 and CYP3A5 [16]. The expression of CYP3A5 is polymorphic and present in around 10–20% [48] of Caucasians where it is correlated with CYP3A4 through a shared mechanism for expression [29]. The latent variable approach was then compared with the classic Sobol’s variance-based GSA, Sobol’s GSA performed by grouping together the correlated factors, and the Kucherenko approach.

## Materials and methods

### Variance-based sensitivity analysis and the Kucherenko approach

Let us consider the generic model in Eq. 1:

1 $$\begin{aligned} Y = f({\mathbf {X}}), \end{aligned}$$

where *Y* is the scalar model output, ${\mathbf {X}}$ is the vector including the *k* independent input factors ($X_i$, $i=1,\ldots ,k$) and *f* is the input–output relationship. In variance-based GSA, two sensitivity indices are derived from the functional decomposition of the variance (*V*) of *Y*, in Eq. 2.

2 $$\begin{aligned} V(Y) = \sum _{i=1}^{k}V_i + \sum _{i}\sum _{j>i}V_{i,j} + \cdots + V_{1,\ldots ,k} \end{aligned}$$

The functional decomposition of the variance presented in Eq. 2 is also known as *functional ANOVA* [50, 51]. $V_i$ is called the first order term and it is the portion of *V*(*Y*) explained by the variation of each $X_i$ taken alone [49], where *E* is the expectation operator. $V_{i,j}$ is the second order term and it is the portion of *V*(*Y*) explained by the interaction between $X_i$ and $X_j$. Similarly, it is possible to define all the higher order interaction terms. Variance-based, or Sobol, sensitivity indices can be defined from 2 as in Eq. 3 [18, 50].

3 $$\begin{aligned} \begin{aligned} S_{i}&= \dfrac{V_i}{V(Y)}= \dfrac{V_{X_i}(E_{\mathbf {X_{\sim i}}} (Y \, | \, X_i))}{V(Y)} \\ S_{Ti}&= \frac{E_{{\mathbf {X}}_{\sim i}} ( V_{X_i}( Y \, | \, {\mathbf {X}}_{\sim i} ) )}{V(Y)} \end{aligned} \end{aligned}$$

$S_i$ is the so called first order index (or main effect) and $S_{T,i}$ is the total effect. ${\mathbf {X}}_{\sim i}$ represents a vector including all the factors except $X_i$. $S_i$ is related with the part of *V*(*Y*) explained by the variation of $X_i$ taken singularly and $S_{T,i}$ is the sum of $S_i$ with all the interaction effects of $X_i$ with the other inputs [49, 50]. When the parameters are independent, the relationships $S_i \le S_{T,i}$ and $\sum S_i \le 1$ are always valid and $S_{T,i}-S_i$ gives information about the extent of interaction effects involving $X_i$ [49, 50].

The GSA method proposed by Kucherenko et al. [24] extends the variance based methods for models with dependent input factors. Here, the main and total effects of the variance-based GSA are calculated with a copula-based method. With this approach, $S_i$ includes the effects of the dependence of $X_i$ with other factors [31] and can be higher than $S_{T,i}$. As reported by [31], $S_{T,i}$ includes only the effects of $X_i$ that are not due to its dependence with ${\mathbf {X}}_{\sim i}$. A given factor whose importance is only due to the correlation with another factor would have $S_{T,i}=0$, but $S_i$ can differ from 0 [31]. Moreover, $S_{T,i}$ approaches 0 as the correlation $|\rho | \rightarrow 1$ [24]. A possible explanation for this behaviour is that as the correlation approaches 1, the value of $X_i$ is completely informed by ${\mathbf {X}}_{\sim i}$ and thus $V_{X_i}( Y \, | \, {\mathbf {X}}_{\sim i} )$ will tend to 0.

### Latent variable approach for GSA

The latent variable approach expresses the inter-correlation between two parameters as causal relationships between uncorrelated variables and therefore, it allows the use of classical variance-based GSA.

**Fig. 1:**
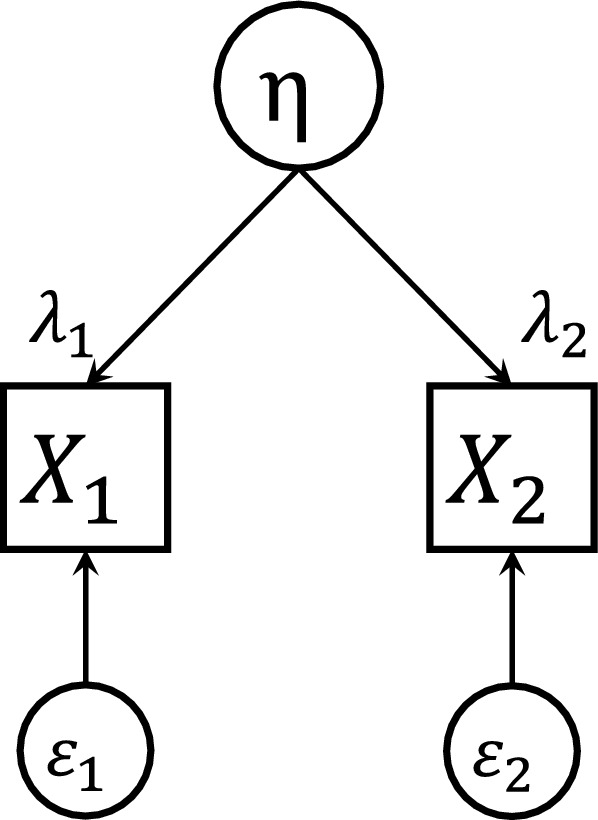
Relationship between the observed, common and unique variances for two correlated parameters and one latent variable. $X_1$ and $X_2$ are the observed variables, $\eta$ is the latent variable, $\varepsilon _1$ and $\varepsilon _2$ are the unique variances and $\lambda$ are the factor loadings

Latent variable methods partition the *observed variance* of each correlated parameter (observed variable) into two parts: a *common variance*, caused by the latent variable and a *unique variance*, specific to the parameter itself [4]. In this work, we focus on the case of two linearly correlated random variables whose correlation is explained by one latent variable. The relationship between the observed, common and unique variances for two correlated parameters and one latent variable is reported through a path diagram as shown in Fig. 1 [28]. Following the notation of latent-variable methodology, $\eta$ is the latent variable, and is conventionally represented by a circle in the path diagram. Unidirectional arrows represent the causal relationships between latent and dependent factors $X_i$, $i=1,2$ (depicted by a box) and $\varepsilon _i$ represents the unique variance associated with $X_i$ [4]. $X_1$ and $X_2$ are considered linearly correlated, with a linear (Pearson) correlation coefficient of $\rho _{12}$. Here we assume that $\eta$, $X_i$ and $\varepsilon _i$ are distributed as in Equation system 4 and that $\eta$ and $\varepsilon _i$ are independent.

4 $$\begin{aligned} \begin{aligned} \eta&\sim {\mathcal {N}} (0, 1) \\ X_i&\sim {\mathcal {N}} (0,1) \\ \varepsilon _i&\sim {\mathcal {N}} \left( 0, \sigma _i^2\right) \end{aligned} \end{aligned}$$

A common assumption is that the causal relationships between $\eta$ and $X_i$ are linear. In this case, it is possible to write the following Equation system 5 [4, 28].

5 $$\begin{aligned} \begin{aligned} X_1&= \lambda _1 \, \eta + \varepsilon _1 \\ X_2&= \lambda _2 \, \eta + \varepsilon _2 \end{aligned} \end{aligned}$$

$\lambda _1$ and $\lambda _2$ are called the *factor loadings* and represent the correlations of $X_1$ and $X_2$ with $\eta$ [14]. Given that our hypothesis is that $\eta$ and $X_i$ are standard normal random variables, and that $\varepsilon _i$ is distributed normally with a mean equal to 0 and variance $\sigma _i^2$, by calculating the variance of both sides of the equations in Equation system 5, it is possible to derive that $\sigma _i^2 = (1 - \lambda _i^2)$, $i=1,2$.

Now, to correctly express $X_1$ and $X_2$ as functions of $\eta$, we need to define $\lambda _1$, $\lambda _2$ and $\sigma _1^2$, $\sigma _2^2$. According to *path analysis* theory, the correlation between $X_1$ and $X_2$ can be expressed as $\rho _{12} = \lambda _1 \cdot \lambda _2$ [28]. With the hypotheses that $\rho _{12}>0$ and that $X_1$ and $X_2$ have the same relationship with $\eta$, thus $\lambda _1=\lambda _2=\lambda$, it is possible to define $\lambda$ as in Eq. 6 [28].

6 $$\begin{aligned} \lambda = \sqrt{\rho _{12}} \end{aligned}$$

Another possible solution is $\lambda =-\sqrt{\rho _{12}}$, where the latent variable has a negative correlation with both $X_1$ and $X_2$. In case of $\rho _{12}<0$, the absolute values of both factors loadings are equal to $\sqrt{\rho _{12}}$, while their signs are opposite.

According to Eq. 5, $\lambda ^2$ is the portion of the variance of $X_i$ that is attributed to the latent factor. With our approach, $\lambda ^2$ is the average variance extracted (AVE). AVE can be defined as *the average amount of variation that a latent construct is able to explain in the observed variables* [14]. Intuitively, this is the overall amount of variance that ‘is taken’ from our dependent factors $X_i$ and attributed to the latent variable $\eta$, in order to define the causal relationships in Eq. 5. As shown in the Appendix 5, with our hypothesis that $X_1$ and $X_2$ have the same relationship with $\eta$, the AVE is minimised. This means that we are explaining the correlation between two observed variables by attributing (on average) the minimum variance possible to the latent construct.

**Table 1:** Assumptions for the use of the latent variable approach

Assumptions^a^	
Only two correlated input factors $X_1$ and $X_2$	
A linear correlation between $X_1$ and $X_2$	
$\eta$, $\varepsilon _1$, $\varepsilon _2$, $X_1$, $X_2$ normally distributed as in Eq. 4	
Linear relation between $\eta$ and $X_1$, $X_2$, as in Eq. 5	
Same relation between $X_1$, $X_2$ and $\eta$, thus $|\lambda _1|=|\lambda _2|=|\lambda |$ in Eq. 5	

^a^$X_1$, $X_2$ are the dependent input factors

$\eta$ is the latent variable

$\varepsilon _1$, $\varepsilon _2$ are the unique variances

With the latent variable approach, instead of two correlated random variables ($X_1$ and $X_2$), three independent random variables ($\eta$, $\varepsilon _1$ and $\varepsilon _2$) will be considered in the variance-based GSA. In this context, the impact of $\varepsilon _1$ and $\varepsilon _2$ on the model output can be uniquely attributed to $X_1$ and $X_2$, respectively. Instead, it would be impossible to distinguish if the impact of $\eta$ on the model output is primarily mediated by $X_1$ or $X_2$.

For simplicity, we have considered standardised variables. However, the latent variable approach can easily be extended to data in original units with the use of simple transformations. Nevertheless, in order to use this method several assumptions must be satisfied (summarised in Table 1) and some limitations still exist. The sums of the random variables representing the latent and independent variances must follow the distributions of $X_i$. This condition is satisfied if both the parameters are normally distributed and it can easily be extended to the case of the two parameters being log-normally distributed. However, the condition in Equation system 5 is not easily satisfied for other types of distributions. The method presented here is valid when considering two correlated factors and it can be extended to three mutually correlated factors, by using the so called *method of triads* to derive a unique solution for the factor loadings [28]. However, it is possible that there is no unique solution when more than three mutually correlated factors are considered [28]. In this situation, the application of the latent variable approach for GSA would become more challenging.

The practical implementation of the latent variable approach is relatively straightforward. First, $\lambda$ is defined as per Eq. 6, where $\rho _{12}$ is the linear correlation between the two variables of interest, $X_1$ and $X_2$. Then, the values for $\eta$ are extracted from a standard normal distribution, while the ones for $\varepsilon _1$ and $\varepsilon _2$ are extracted from a normal distribution, with mean 0 and variance $\sigma ^2=1-\lambda ^2$. $X_1$ and $X_2$ are then defined as per Eq. 5. By doing this, $X_1$ and $X_2$ would be standard normal random variables. Then, they can be easily transformed to normal variables with the desired mean and standard deviation. As previously stated, the approach can be extended to $X_1$ and $X_2$ being log-normally distributed, although in this case $\log (X_1)$ and $\log (X_2)$ should be linearly correlated.

### Algebraic models

The latent variable approach was initially tested on three algebraic models, namely model 1, 2 and 3, in Eqs. 7, 8 and 9 respectively.

7 $$\begin{aligned} Y= & {} X_1 + X_2 + X_2 \cdot X_3 \end{aligned}$$

8 $$\begin{aligned} Y= & {} X_1 + X_2 + X_1 \cdot X_3 \end{aligned}$$

9 $$\begin{aligned} Y= & {} X_1 + X_2 + X_3 + X_4 \end{aligned}$$

For all the models, all factors were considered to be normally distributed with means equal to 0 and variances equal to 1, $X_i \sim {\mathcal {N}} (0,1)$, $i=1,2,3,4$. $X_1$ and $X_4$ were considered linearly correlated, with a Pearson correlation coefficient of $\rho _{14}$. Model 1 and model 2 differ in the fact that in model 1, $X_1$ is not involved in any interaction, while in model 2, $X_1$ interacts with $X_3$.

$X_4$ does not appear in the model 1 or model 2 equations, consequently, its ‘causal impact’^1^ on the model output *Y* must be null. Intuitively, for both model 1 and 2, the results of a variance-based GSA in absence of correlation, considering only $X_1$, $X_2$ and $X_3$, will correctly reflect the structure of the model.

### Whole-body PBPK model for midazolam

**Fig. 2:**
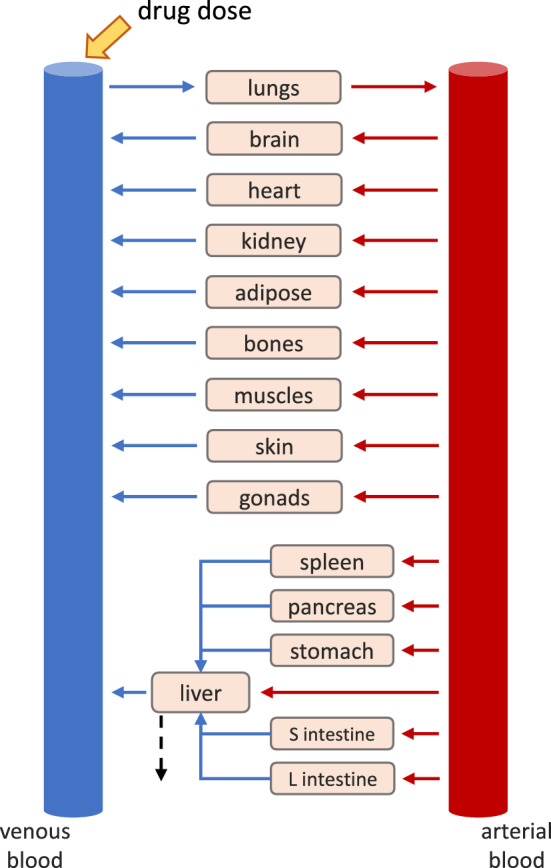
Structure of a general whole-body PBPK model. Each box corresponds to a specific compartment. The red and blue arrows represent the arterial and venous blood flow, respectively. The black-dashed arrow represents elimination through metabolism in the liver. The yellow arrow represent the drug intravenous administration. *S intestine* and *L intestine* are the small and large intestine, respectively

A whole-body PBPK model was developed, describing the pharmacokinetics of the drug MDZ following an intravenous (IV) bolus injection in a population of human healthy subjects. The model is represented in Fig. 2. This section provides a brief description of the model. For a detailed account of the model equations, the parameters used for the PBPK construction and the algorithm used for generating the population, see the Supplementary Material.

The typical equation used to describe the mass balance in a given organ or tissue *t* within a PBPK model is reported in Eq. 10. For a detailed description and the underlying theories of this model, called *well-stirred perfusion-limited* PBPK, please refer to [2].

10 $$\begin{aligned} \frac{dx_t}{dt} = Q_t \, \biggl ( \frac{x_{art}}{V_{art}} - \frac{x_t/V_t}{P_{t:p}/B:P} \biggr ) \end{aligned}$$

Equation 10 is valid for all organs and tissues except the liver, the lungs, the arterial and venous blood. $x_t$ is the drug amount in compartment *t*, while $V_t$ is the volume. Subscript *art* stands for arterial blood. $Q_t$ is the blood flow to compartment *t*. *B* : *P* is the blood-to-plasma ratio and $P_{t:p}$ is the tissue-to-plasma partition coefficient.

MDZ is primarily metabolised in the liver by the two enzymes, CYP3A4 and CYP3A5. For MDZ both enzymes catalyse two reactions, leading to the formation of two metabolites,*1-hydroxy* *midazolam* (1-OH-MDZ) and *4-hydroxy* *midazolam* (4-OH-MDZ) [16, 54]. For this reason, two mass flows corresponding to MDZ metabolism leave the PBPK system from the liver compartment, as represented in Eq. system 11.

11 $$\begin{aligned} \begin{aligned} \frac{dx_{liv}}{dt}&= Q_{liv} \biggl (\frac{x_{art}}{V_{art}} - \frac{x_{liv}/V_{liv}}{P_{liv:p}/B:P}\biggr ) + \sum _{t \in {\mathcal {S}}} \Biggl [ Q_t \, \biggl ( \frac{x_t/V_t}{P_{t:p}/B:P} \biggr ) \Biggr ] \\&\quad - MET_{3A4} - MET_{3A5} \end{aligned} \end{aligned}$$

Subscript *liv* stands for liver, ${\mathcal {S}}$ represents the splanchnic organs (spleen, pancreas, stomach, small and large intestine). $c_{u,liv}$ is the unbound liver concentration. $MET_{3A4}$ and $MET_{3A5}$ are the fluxes representing the reactions catalysed by CYP3A4 and CYP3A5. All the chemical reactions are described using *Michaelis–Menten* equations [39]. The *Michaelis–Menten* parameters for MDZ are taken from in vitro studies [16] and they are scaled to the in vivo context as per [46]. One of the main parameters used for the in vitro to in vivo scaling is the microsomal protein per gram of liver (*MPPGL*) (see supplementary materials for a detailed description of this process).

The population variability of physiological parameters such as the compartment volumes and blood flow was generated with a simple algorithm having as inputs the sex of the subject, the height and the body mass index (BMI).

To simulate an IV bolus injection of 5 mg of MDZ, the initial condition of the venous blood compartment was set equal to 5, while the remaining compartments were set to equal 0. The *area under the curve* (AUC) of the venous plasma compartment from time 0 to $24\cdot 7$ *h* was considered the output of interest for the GSA. The distributions of the model parameters considered in this analysis are reported in Table 2.

**Table 2:** Variable parameters used for the MDZ PBPK model

Parameters	Distribution parameters	Distribution type	Units	References
Sex^d^	0, 1			
height (male)^e^	176.7 (6.15)	Normal^a^	cm	[5]
Height (female)^e^	163.3 (5.85)	Normal^a^	cm	[5]
BMI^f^	18.5, 24.9	Uniform^b^	$kg/m^2$	[55]
$[CYP3A4]^g$	137 (41%)	log-normal^c^	$(pmol\,CYP)/(mg\,MP)$	[8]
$[CYP3A5]^g$	103 (65%)	log-normal^c^	$(pmol\,CYP)/(mg\,MP)$	[8]
$MPPGL^h$	39.79 (27%)	log-normal^c^	$(mg\,prot)/(g\,liver)$	[30]

^a^For distribution parameters, *mean (standard deviation)* of the normal variable

^b^For distribution parameters, *minimum*, *maximum* of the parameter

^c^For distribution parameters, *mean (CV)* of the log-normal variable

^d^Samples from an uniform distribution are extracted: if the extracted value is $<0.5$ the subject is female (0), otherwise male (1)

^e^Height for a 20 years old Italian population

^f^Body mass index corresponding to the nutritional status of ‘Normal weight’ according to the World Health Organization

^g^CYP abundance per mg of microsomal protein

^h^ mg of microsomal proteins for gram of liver

### Analysis overview

For the GSA, the following methods were applied to both the algebraic and the PBPK models:

- classical variance-based GSA considering all the parameters uncorrelated;
- variance-based GSA grouping together the two correlated parameters;
- the method developed by Kucherenko for computing the variance-based GSA indices in presence of correlation [24];
- the latent variable approach.

Concerning the algebraic models, the analysis was carried out varying $\rho _{14}$, from − 0.9 to 0.9. When $\rho _{14}>0$, the latent variable was considered to be positively correlated with both $X_1$ and $X_4$ ($\lambda >0$). Instead, when $\rho _{14}<0$, the latent variable was considered to be positively correlated with $X_1$ and negatively correlated with $X_4$.

For the PBPK model, the (Pearson) correlation between the logarithms of CYP3A4 and CYP3A5 abundances $\rho _{3A4,3A5}$ was considered to equal 0.52, based on proteomic data from human liver samples [1], for the variance-based GSA with grouped factors, for the Kucherenko and the latent variable approaches. In this analysis, all simulated individuals were assumed to express CYP3A5.

All analysis was performed in MATLAB R2020a^2^ [33]. The systems of differential equations were solved with the ode15s MATLABsolver, for a timespan ranging from 0 to $24\cdot 7$ *h*. GSA was performed using the software UQLab [32] except for the variance-based GSA with groups, where an ‘ad hoc’ MATLAB code was developed. For the numerical estimation of the sensitivity indices, within UQLab, the *homma* estimator was used for the Sobol approach, while the default estimator embedded in the software was used for the Kucherenko approach. Concerning the ‘ad hoc’ MATLAB code, we used the estimator reported in [50] (the *errata corrige* version). For all the methods, the sample size was fixed to 10,000. The uncertainty of the sensitivity indices estimates was assessed by using 1000 bootstrap samples, with the exception of the Kucherenko method, where the convergence plots were used.

## Results

### Algebraic models

**Fig. 3:**
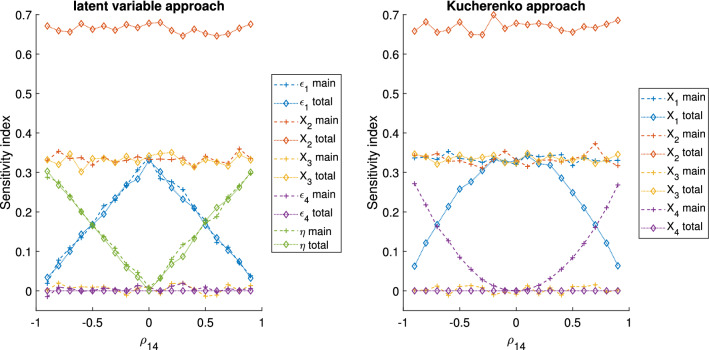
Algebraic model 1 GSA results of the latent variable and the method presented by Kucherenko 2012 [24]

**Fig. 4:**
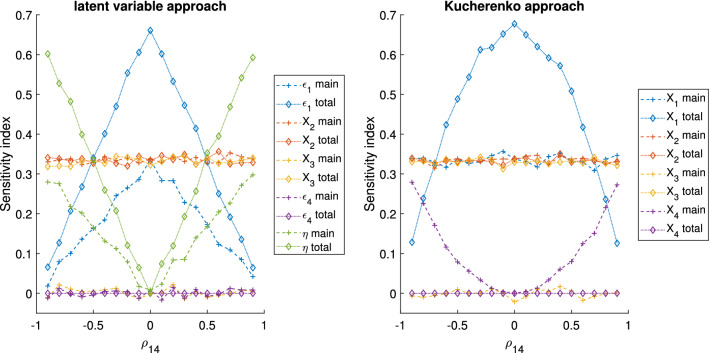
Algebraic model 2 GSA results of the latent variable and the method presented by Kucherenko 2012 [24]

**Fig. 5:**
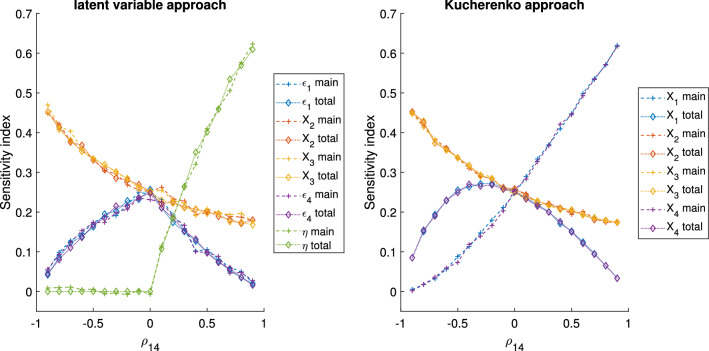
Algebraic model 3 GSA results of the latent variable and the method presented by Kucherenko 2012 [24]

The GSA results for the algebraic models 1, 2 and 3, with $\rho _{14}=0.7$ and $\rho _{14}=0.9$, are reported in Tables 3, 4 and 5, respectively. In Fig. 3 the GSA results obtained with the latent variable and the Kucherenko approaches for the algebraic model 1 are given as a function of $\rho _{14}$, ranging from − 0.9 to 0.9. For the models 2 and 3, the equivalent information is shown in Figs. 4 and 5, respectively. Here we begin by reporting the results of model 1 and 2 and then, model 3.

The parameter $X_4$ does not appear in Eqs. 7 and 8. Regardless of presence or absence of correlation between $X_1$ and $X_4$ its ‘causal’ impact on the output should therefore be null. Hence, intuitively, the results of a variance-based GSA with the classic Sobol’s method considering only $X_1$, $X_2$ and $X_3$ should be the ones that truly represent the model structure. Any differences in main and total effects for the Kucherenko approach, the latent variable approach and the variance based GSA with grouped factors are therefore due to how these methods handle the correlation.

Concerning the Kucherenko approach, in Fig. 3 the higher the absolute value of $\rho _{14}$ is, the higher the main effect of $X_4$ is, while its total effect always remains equal to 0. This substantially confirms the findings of [31], where it was highlighted that an input whose importance is due to the dependencies with other inputs has a total effect equal to 0, but a main effect that can be higher than 0. Moreover, as the absolute value of the correlation increases, the total effect of $X_1$ decreases, while the main effect remains stable. From [31] we know that $S_1$ includes the impact of the correlation of $X_1$ with $X_4$, while $S_{T,1}$ just includes the ‘uncorrelated’ effects. From our example is possible to appreciate that the higher $|\rho _{14}|$ is, the lower the ‘uncorrelated’ effect of $X_1$ is. In this context it is actually challenging to distinguish between the ‘causal’ effect of $X_1$ and $X_4$ on *Y* and the effect due to their dependence. Similar conclusions can be made for the model 2. By limiting the analysis to the Kucherenko indices, it is challenging to understand how much $X_1$ is involved in interaction effects and, ultimately, to determine any ranking of importance of the parameters as can be used in practical applications.

Concerning the latent variable approach, presented in Figs. 3 and 4, the higher the absolute value of $\rho _{14}$ is, the higher the importance of the latent variable over the unique variances. Ultimately, with $\rho _{14}$ approaching 1 the whole variance of both $X_1$ and $X_4$ becomes fully explained by the latent factor and thus, the residual variances’ effect on the output variance tends to 0. Given that the latent variable affects both the correlated factors equally, it is not possible to elucidate if the impact of $\eta$ on the output variance is primarily mediated by $X_1$ or $X_4$. However, the impact of the unique variances can be uniquely attributed to the correlated factors. In fact, for both models 1 and 2, both the main and total effect of $\varepsilon _4$ are always equal to zero, as seen in Figs. 3 and 4. This is unlikely the case for traditional variance-based GSA with groups (see Tables 3 and 4), where, independently of the values of $\rho _{14}$, it is not possible to determine the impact of the variable within the groups. Notably, if $|\rho |$ is close to 1, the latent variable will fully explain both $X_1$ and $X_4$, resembling the case of the grouping approach. Given that in both the grouping and the latent variable approach we are performing a standard Sobol’s GSA with uncorrelated factors, the interpretation of the sensitivity indices and the factor ranking is straightforward.

In model 1, $X_1$ is not involved in any interactions. This is discernible when $S_i=S_{T,i}$. In this case, $S_1=S_{T,1}$, as seen in Table 3 and Fig. 3. Neither $\eta$ or $\varepsilon _1$ are involved in any interactions. This is quite intuitive as the model is linear and $X_1$ is defined as the sum of the latent variable and the unique variance in the latent variable approach. However, interaction effects between the latent variable and the unique variance will arise, for example, in case of $X_1$ having a nonlinear effect (e.g., quadratic) on *Y*^3^. In model 2, $X_1$ and $X_3$ show interaction effects, as noted in the Sobol’s GSA results. This happens when $S_{T,i}>S_i$. In Table 4 and Fig. 4 we can see that both the latent variable and the unique variance of $X_1$ show interaction effects.

Concerning model 3, Table 5 and Fig. 5, we observe that the sensitivity indices of $X_2$ and $X_3$ change in function of $\rho _{14}$. The traditional variance-based GSA that considers all the factors uncorrelated does not capture this effect. With this simple example, we can see that ignoring the correlation within GSA could potentially bias the overall results of the analysis. Traditional GSA with groups can capture this effect and thus, it can be an easy and reliable method for treating correlations. However, as explained for models 1 and 2, it has the limitation of not distinguishing the impact of the variables within the groups of correlated factors.

Concerning the Kucherenko approach, $S_1$ and $S_4$ are close to 0 when $\rho _{14}$ is close to -1 and they both grow as $|\rho _{14}|$ grows. Instead, $S_{T,1}$ and $S_{T,4}$ have almost a parabolic shape. Both the main and total effects of $X_1$ and $X_4$ are low for strong negative correlation, probably because in this model the effect of $X_1$ tends to cancel the one of $X_4$ on *Y* and vice versa. For a high positive correlation the total effects tend to zero, while the main effects are close to 0.6.

Regarding the latent variable approach, one interesting observation is that the overall tendency of the unique variances and latent variable sensitivity indices are similar to those of the total and main effects of $X_1$ and $X_4$ of the Kucherenko approach, respectively. This probably happens because the unique variances represents the impact of the ‘uncorrelated’ part of the factors, similarly to the total effect of the Kucherenko approach. Instead, both the latent variable and the main effect include the ‘dependent’ part of the factors. However, one important difference is that the latent variable approach is a variance-based GSA performed with independent variables and thus, the indices are easily understandable, this is unlikely the case for the Kucherenko approach. Finally, it is interesting to observe that for negative correlations the impact of the latent variable is zero. This happens because the factor loadings ($\lambda$) are equal in module, but opposite in sign and thus, the latent variable term is cancelled from Eq. 9.

**Table 3:** Sensitivity indices for the algebraic model 1

	Sobol^a^		Kucherenko^b^		Latent variable^a^		Grouped^a^	
Factor	Main	Total	Main	Total	Main	Total	Main	Total
			$$\rho _{14}=0.7$$					
$X_1^c$	0.34	0.33	0.33	0.17	0.11	0.1	0.31^d^	0.34^d^
	(0.32,0.36)	(0.31,0.34)			(0.09,0.13)	(0.9,0.11)	(0.29,0.33)	(0.31,0.37)
$X_2$	0.33	0.67	0.32	0.64	0.32	0.65	0.31	0.7
	(0.31,0.35)	(0.64,0.7)			(0.3,0.35)	(0.63,0.67)	(0.28,0.33)	(0.67,0.72)
$X_3$	0	0.33	0	0.34	0.02	0.33	− 0.03	0.32
	(− 0.03,0.02)	(0.31,0.35)			(− 0.01,0.04)	(0.31,0.35)	(− 0.06,0)	(0.29,0.34)
$X_4^c$	0	0	0.16	0	0.02	0		
	(− 0.02,0.02)	(0,0)			(0,0.03)	(0,0)		
$\eta$					0.26	0.23		
					(0.24,0.28)	(0.22,0.25)		
			$$\rho _{14}=0.9$$					
$X_1^c$	0.33	0.35	0.33	0.06	0.05	0.04	0.33^d^	0.34^d^
	(0.31,0.35)	(0.33,0.37)			(0.03,0.07)	(0.03,0.04)	(0.31,0.35)	(0.31,0.37)
$X_2$	0.32	0.66	0.33	0.69	0.33	0.65	0.35	0.67
	(0.29,0.34)	(0.64,0.69)			(0.31,0.35)	(0.63,0.68)	(0.33,0.38)	(0.64,0.7)
$X_3$	− 0.01	0.33	− 0.01	0.35	0.02	0.35	0	0.33 (0.31,0.36)
	(− 0.04,0.02)	(0.31,0.36)			(− 0.01,0.04)	(0.33,0.37)	(− 0.03,0.02)	(− 0.03,0.02)
$X_4^c$	− 0.01	0	0.27	0	0.01	0		
	(− 0.03,0.01)	(0,0)			(− 0.01,0.03)	(0,0)		
$\eta$					0.3	0.29		
					(0.28,0.32)	(0.27,0.3)		

^a^Values reported in the table are mean (2.5,97.5) percentiles calculated with 1000 bootstrap samples

^b^Convergence plots are shown in the supplementary materials

^C^For the latent variable model, this refers to the unique variance

^d^This refers to the $X_1$ and $X_4$ group

**Table 4:** Sensitivity indices for the algebraic model 2

Factor	Sobol^a^		Kucherenko^b^		Latent variable^a^		Grouped^a^	
	Main	Total	Main	Total	Main	Total	Main	Total
			$$\rho _{14}=0.7$$					
$X_1^c$	0.34	0.68	0.32	0.34	0.11	0.2	0.33^d^	0.68^d^
	(0.32,0.36)	(0.66,0.71)			(0.09,0.13)	(0.18,0.21)	(0.3,0.35)	(0.65,0.71)
$X_2$	0.32	0.33	0.33	0.32	0.33	0.33	0.32	0.34
	(0.3,0.34)	(0.32,0.35)			(0.31,0.35)	(0.31,0.35)	(0.3,0.34)	(0.31,0.37)
$X_3$	0	0.34	0	0.34	0.01	0.33	− 0.03	0.33
	(− 0.02,0.03)	(0.32,0.36)			(− 0.01,0.04)	(0.3,0.35)	(− 0.05, − 0.01)	(0.31,0.35)
$X_4^c$	− 0.01	0	0.16	0	0.01	0		
	(− 0.03,0.01)	(0,0)			(− 0.01,0.02)	(0,0)		
$\eta$					0.24	0.47		
					(0.22,0.26)	(0.45,0.49)		
			$$\rho _{14}=0.9$$					
$X_1^c$	0.33	0.66	0.32	0.13	0.03	0.06	0.36^d^	0.65^d^
	(0.31,0.35)	(0.63,0.69)			(0.01,0.05)	(0.05,0.07)	(0.33,0.38)	(0.62,0.68)
$X_2$	0.32	0.34	0.32	0.33	0.32	0.33	0.35	0.32
	(0.3,0.34)	(0.32,0.35)			(0.3,0.34)	(0.32,0.35)	(0.33,0.37)	(0.29,0.35)
$X_3$	0	0.34	0	0.35	− 0.01	0.34	0.01	0.34
	(− 0.03,0.03)	(0.32,0.37)			(− 0.03,0.01)	(0.32,0.37)	(− 0.01,0.04)	(0.32,0.37)
$X_4^c$	0	0	0.25	0	0	0		
	(− 0.02,0.02)	(0,0)			(− 0.02,0.02)	(0,0)		
$\eta$					0.29	0.61		
					(0.27,0.32)	(0.59,0.64)		

^a^Values reported in the table are mean (2.5,97.5) percentiles calculated with 1000 bootstrap samples

^b^Convergence plots are shown in the supplementary materials

^c^For the latent variable model, this refers to the unique variance

^d^This refers to the $X_1$ and $X_4$ group

**Table 5:** Sensitivity indices for the algebraic model 3

Factor	Sobol^a^		Kucherenko^B^		Latent variable^a^		Grouped^a^	
	Main	Total	Main	Total	Main	Total	Main	Total
			$$\rho _{14}=0.7$$					
$X_1^c$	0.25	0.25	0.55	0.1	0.07	0.05	0.62^d^	0.63^d^
	(0.23,0.26)	(0.23,0.26)			(0.05,0.09)	(0.04,0.06)	(0.6,0.64)	(0.61,0.65)
$X_2$	0.25	0.24	0.19	0.19	0.18	0.19	0.19	0.18
	(0.23,0.26)	(0.23,0.26)			(0.16,0.2)	(0.18,0.2)	(0.17,0.21)	(0.16,0.2)
$X_3$	0.26	0.25	0.18	0.19	0.2	0.19	0.18	0.19
	(0.24,0.27)	(0.24,0.27)			(0.18,0.22)	(0.18,0.2)	(0.16,0.2)	(0.17,0.2)
$X_4^c$	0.26	0.25	0.54	0.1	0.06	0.05		
	(0.24,0.28)	(0.24,0.27)			(0.04,0.08)	(0.05,0.06)		
$\eta$					0.51	0.51		
					(0.49,0.53)	(0.49,0.53)		
			$$\rho _{14}=0.9$$					
$X_1^c$	0.24	0.25	0.63	0.03	0.02	0.02	0.65^d^	0.65^d^
	(0.22,0.26)	(0.24,0.26)			(0,0.04)	(0.02,0.02)	(0.63,0.67)	(0.63,0.67)
$X_2$	0.24	0.25	0.17	0.17	0.18	0.17	0.17	0.17
	(0.22,0.26)	(0.24,0.26)			(0.16,0.2)	(0.16,0.18)	(0.15,0.19)	(0.15,0.19)
$X_3$	0.26	0.24	0.18	0.17	0.17	0.17	0.18	0.19
	(0.24,0.28)	(0.23,0.25)			(0.15,0.19)	(0.16,0.18)	(0.16,0.2)	(0.17,0.21)
$X_4^c$	0.25	0.26	0.62	0.03	0.02	0.02		
	(0.23,0.27)	(0.25,0.28)			(0,0.04)	(0.01,0.02)		
$\eta$					0.63	0.62		
					(0.61,0.64)	(0.6,0.64)		

^a^Values reported in the table are mean (2.5,97.5) percentiles calculated with 1000 bootstrap samples

^b^Convergence plots are shown in the supplementary materials

^c^For the latent variable model, this refers to the unique variance

^d^This refers to the $X_1$ and $X_4$ group

### Whole-body PBPK model for midazolam

The simulated MDZ plasma concentration-time profiles and AUCs for a population of 10,000 subjects are shown in Supplementary Figs. 8 and 9, respectively. The GSA results of Sobol’s method without accounting for the correlation, of the Kucherenko method, of the traditional variance-based GSA with groups and of the latent variable approach are presented in Table 6.

According to the results from Sobol’s GSA, the most important parameters in explaining the variability in AUC are (in order of importance) the MPPGL, CYP3A4 and CYP3A5 abundances. These factors are important because they control the rate of metabolism in the liver. The fact that the metabolism-related parameters are the most important for explaining variability in AUC suggests that the rate-limiting step of drug elimination is the metabolism and not, for example, liver blood flow. Given that exposure drives drug effect, the interindividual variability in efficacy, due to PK, is mainly explained by genetics in this case example. However, we need to consider that our population is composed by healthy adults with a BMI corresponding to the nutritional status of ‘normal weight’ [55]. The inclusion of overweight or obese subjects may impact the results of the GSA.

Concerning the GSA results obtained with the Kucherenko, the variance-based GSA with groups and the latent variable approach, the sensitivity indices of MPPGL are slightly reduced as compared to Sobol’s GSA. This is most likely related with the fact that the correlation between CYP3A4 and CYP3A5 tends to generate more ‘extreme’ individuals, i.e., poor metabolisers (with low CYP3A4 and low CYP3A5 abundances) and rapid metabolisers (with high CYP3A4 and high CYP3A5 abundances). Thus, as it is possible to observe in Supplementary Fig. 9, the AUC distribution in case of correlation is slightly wider with respect to the case of no correlation. These results are in agreement with our previous studies, where we showed how a positive correlation between two enzymes metabolising a given compound can cause a widening of the systemic AUC distribution [37].

Concerning the Kucherenko analysis, it is difficult to confidently use either the main or the total effects for the purpose of factor ranking. For example, by observing the main effect the two most important parameters are CYP3A4 and CYP3A5 abundances. However, it is difficult to understand what the contributions of the variables themselves are and what is due to the correlation. For this reason, in our example, there is a risk of overestimating the importance of the enzymatic abundances and, by extension, underestimating the importance of the other factors. By using the total effect for the factor ranking, there is instead the risk of underestimating the importance of the correlated factors and overestimating the importance of the remaining inputs, as the total effects for the factors involved in the correlation tend to 0 as $|\rho | \rightarrow 1$ [24]. Moreover, by using these two indices, given that for both CYP3A4 and CYP3A5 abundances the total effect is lower than the main effect, it is difficult to understand the effect of interactions.

In the latent variable approach, the factor ranking can be done by examining either the main or at the total effects. This is possible because the correlation between CYP3A4 and CYP3A5 was expressed in terms of a functional relationship between three independent factors, the latent variable and two independent variances. Thus, the classical variance-based GSA was used. With this approach, the most important factor in explaining the AUC is $\eta$, followed by MPPGL and the independent components of CYP3A4 and CYP3A5. By using either the main or the total effect for the factor ranking, we can confidently assess that the main drivers for the plasma AUC are the metabolism-related parameters. Moreover, with this method it is possible to appreciate the interaction effects, that in this case are mild and do not have a great impact on the factor ranking. A downside of this approach is that $\eta$ drives both CYP3A4 and CYP3A5 variability. For this reason, given that the latent variable is one of the two most important parameters, it is not possible to appreciate if its importance is primarily caused by the CYP3A4 or CYP3A5 mediated pathway. By investigating the independent components of CYP3A4 and CYP3A5 abundances, it is noted that they do have a similar impact. Intuitively, if one of the two factors was not important for the AUC, the independent component would be equal to zero (however, it is not necessarily true for the opposite case).

The results of the PBPK simulations presented here aim to illustrate a GSA methodology, only. Therefore, we do not recommend their use for other purposes.

**Table 6:** Sensitivity indices for the MDZ PBPK model

Factor	Sobol^a^		Kucherenko^b^		Latent variable^a^		Grouped^a^	
	Main	Total	Main	Total	Main	Total	Main	Total
			$$\rho _{3A4,3A5}=0.52$$					
sex	0	0.02	0.01	0.02	0.03	0.02	0	0.01
	(− 0.02,0.02)	(0.01,0.03)			(0.01,0.05)	(0.01,0.02)	(− 0.02,0.02)	(− 0.03,0.04)
height	0.01	0.05	0.02	0.03	0.04	0.03	0.01	0.01
	(− 0.01,0.03)	(0.04,0.05)			(0.02,0.06)	(0.02,0.04)	(− 0.01,0.03)	(− 0.02,0.05)
BMI	0.03	0.05	0.03	0.03	0.04	0.03	0.01	0.03
	(0.01,0.05)	(0.04,0.06)			(0.02,0.07)	(0.02,0.05)	(− 0.01,0.03)	(− 0.01,0.06)
MPPGL	0.29	0.39	0.25	0.3	0.26	0.3	0.24	0.29
	(0.27,0.31)	(0.37,0.41)			(0.24,0.29)	(0.27,0.32)	(0.22,0.27)	(0.26,0.32)
CYP3A4^c^	0.27	0.33	0.49	0.22	0.12	0.15	0.61^d^	0.69^d^
	(0.25,0.3)	(0.31,0.35)			(0.1,0.15)	(0.13,0.17)	(0.58,0.64)	(0.67,0.72)
CYP3A5^c^	0.23	0.29	0.42	0.15	0.09	0.1		
	(0.2,0.25)	(0.27,0.31)			(0.07,0.09)	(0.09,0.12)		
$\eta$					0.43	0.48		
					(0.41,0.46)	(0.46–0.5)		

^a^Values reported in the table are mean (2.5,97.5) percentiles calculated with 1000 bootstrap samples

^b^Convergence plots are shown in the supplementary materials

^c^For the latent variable model, this refers to the unique variance

^d^Refers to the group of CYP3A4 and CYP3A5

## Discussion

GSA is gaining use in modelling for pharmaceutics, especially in the field of PBPK M&S. Recent applications in the literature [10, 26, 34, 36–38, 58, 60] and regulatory discussions [6, 7] have indicated the usefulness of these methods and it is likely that GSA will become an important feature of modelling in pharmaceutical R&D and for regulatory decision-making. This development is welcomed, indeed in the field of toxicology GSA is an important part of best practices for risk assessment of dose metric predictions [19, 34, 35, 41].

In order for GSA to gain wider use, the issues of usability and interpretation of the results need to be considered. PBPK M&S is an interdisciplinary effort highly reliant on experts in several domains, including medicinal chemistry, in vitro drug metabolism, pharmacokinetics, pharmacology, toxicology, statistical and mathematical modelling, and more. Further, modelling activities are an important tool for supporting a wide variety of decisions in R&D and regulatory submissions. For this reason, dedicated user-friendly software platforms are widely used [13], facilitating standardisation and easy access for non-expert users. We suspect that this is likely to hold true across many different domains, and therefore relevant across areas of application. In this context, particular attention in communicating GSA results should be paid.

Most whole-body PBPK models include several sets of correlated parameters, many of which constrain the models to realistic parameter combinations. It is therefore important that these correlations are accounted for when performing GSA. Several GSA methodologies have been proposed to account for dependent inputs [11, 24, 31, 52, 57] and the method developed by Kucherenko was proposed for PBPK models [27]. However, considerable debate is still ongoing amongst GSA practitioners on how to appropriately interpret the outcomes of these methods. We believe that the use of methodologies whose interpretation is still a matter of debate, require appropriate care in cases where GSA is called upon to support critical decisions, such as those relating to patient safety. The use of such methods may in fact lead to results that are uninterpretable, or, even worse, open to misinterpretation by non-expert GSA users. Certain applications of PBPK M&S require reliable, robust, well-characterised and tested models [45]. We believe that these requirements should apply for GSA methods and algorithms as well.

Here we propose a relatively simple method using a latent variable approach that deals with correlated input variables in variance-based GSA. The method expresses the correlation between two factors as causal relationships between a latent factor, $\eta$, and two unique variances. As a result this allows the use of classical Sobol’s GSA with uncorrelated factors. In our opinion, the approach provides an intuitive process for implementation and interpretation as illustrated in the analysis for MDZ. By ranking the factors according to the total effects of Sobol’s GSA, it was possible to clearly interpret the sensitivity indices. This allows insights into the model behaviour and to understand what the main drivers of variability are in a given output. By having a unique, easy and universally recognised interpretation of the sensitivity indices, it is possible to use GSA for supporting decision-making with increased confidence.

One of several alternatives to the latent variable approach would be the use of traditional variance-based GSA with groups. The main advantage is that this method allows treating more than two, or three, dependent factors and other dependencies than the linear correlations. However, as highlighted in the results section, with this approach is not possible to separately distinguish the impact of the dependent variables within a given group. Another alternative could be to assign causal dependencies between the correlated factors as we have done in a previous study in the context of PBPK models [37]. However, by doing so to describe the dependency, this will affect the relative significance of one input over the other. The potentially arbitrary choice of assigning dependency will increase the importance of the independent variable in the GSA and may produce misleading results. With the latent variable approach we renounce any attempt to completely distinguish the impact of the two correlated inputs on a given model output. Instead, we highlight the impact of the latent variable $\eta$ (as the ‘common cause’) along with the independent part.

Here we also attempt to examine the shortcomings of the latent variable approach. In fact, the method presents some limitations with regards to the number and the distribution of the factors that are mutually correlated, as described in section 2. Moreover, the results of the latent variable approach need to be interpreted in light of the assumptions summarised in Table 1. In case one or more of these assumption are not satisfied (e.g., for bespoke PBPK platforms), the use of traditional GSA with groups is likely a better choice. Despite this, we believe that the latent variable approach can be of use. In conclusion, further research should be performed to find a reliable and interpretable method for handling multiple correlated inputs in GSA. This can be achieved, for example, by overcoming the current limitations of the latent variable approach to expand its use to more than two or three correlated input factors per latent variable. Alternatively, a clear and universally recognised interpretation should be agreed for more general GSA methods for dependent inputs, such as the approaches proposed by Kucherenko et al. [24] and Mara et al. [31].

### Supplementary Information

Below is the link to the electronic supplementary material.

Supplementary material 1 (pdf 1407 KB)

## Appendix: Average variance extracted

The general AVE expression, corresponding to one latent variable and *k* unique variances, is reported in Eq. 12 [15]. Considering that $\sigma _i^2=1-\lambda _i^2$, AVE can be calculated as the average of the squares of the factor loadings associated with the latent variable [14].

12 $$\begin{aligned} AVE = \frac{ \sum _{i=1}^{k} \lambda _i^2 }{ \sum _{i=1}^{k} \lambda _i^2 + \sum _{i=1}^{k} \sigma _i^2} = \frac{1}{k} \sum _{i=1}^{k} \lambda _i^2 \end{aligned}$$

Considering that in our case $k=2$ (two dependent factors) and $\lambda _1 \cdot \lambda _2 = \rho$, we can derive the expression in Eq. 13.

13 $$\begin{aligned} AVE = \frac{1}{2}(\lambda _1^2 + \lambda _2^2) = \frac{1}{2}\biggl ( \lambda _1^2 + \frac{\rho _{12}^2}{\lambda _1^2} \biggr ) \end{aligned}$$

If we calculate the first derivative of AVE over $\lambda _1$ and set it equal to zero, we can obtain the following expression in Eq. 14.

14 $$\begin{aligned} \begin{aligned} \frac{dAVE}{d\lambda _1}&= \lambda _1 - \frac{\rho _{12}^2}{\lambda _1^3}=0 \\ \lambda _1&= \sqrt{|\rho _{12}|} \\ \lambda _2&= sign(\rho _{12})\cdot \sqrt{|\rho _{12}|} \end{aligned} \end{aligned}$$

Where, $sign(\rho _{12})$ is equal to +1 if $\rho _{12}>0$, while it is equal to -1 if $\rho _{12}<0$. If we calculate the second derivative we can see that it is always positive, thus $|\lambda _1| = |\lambda _2|$ corresponds to a minimum.

## Footnotes

1 Here we refer to ‘causal impact’ as the impact of an input factor $X_i$ on the model output *Y* that is not due to the dependence of $X_i$ with other factors.

2 The codes are made available at the following link https://github.com/NicolaMelillo/latent_variable_GSA.

3 If $X_1=\lambda \eta + \varepsilon$ and $Y=X_1^2$, it is straightforward to derive that $Y=\lambda ^2 \, \eta ^2 + \varepsilon ^2 + 2 \lambda \eta \varepsilon$. In this case, there are interaction effects between $\eta$ and $\varepsilon$.
