# Supplementary material for: A latent variable approach to account for correlated inputs in global sensitivity analysis
Source: J Pharmacokinet Pharmacodyn. 2021 May 25;48(5):671–86. doi: 10.1007/s10928-021-09764-x (PMC8405496; doi:10.1007/s10928-021-09764-x)
Supplement: Supplementary file 1 — Supplementary material 1 (pdf 1407 KB) [file 10928_2021_9764_MOESM1_ESM.pdf]

A latent variable approach to account for correlated inputs in global sensitivity analysis

Supplementary Material

Nicola Melillo & Adam S. Darwich*

1 Physiologically based pharmacokinetic (PBPK) model for midazolam

The typical equation used to describe the mass balance in a given organ or tissue t within a physiologically based pharmacokinetic (PBPK) model is reported in Equation 1.

$$\frac{dx_t}{dt} = Q_t \left(\frac{x_{art}}{V_{art}} - \frac{x_t/V_t}{P_{t:p}/B:P} \right) \tag{1}$$

Equation 1 is valid for all organs or tissues except the liver, the lungs, the arterial and venous blood. x_t is the drug amount in compartment t, while V_t is the volume. Subscript art denotes arterial blood. Q_t is the blood flow to compartment t. $B:P$ is the blood-to-plasma ratio, that is an experimentally derived parameter representing the whole blood drug concentration, divided by the plasma drug concentration at steady state. $P_{t:p}$ is the tissue-to-plasma partition coefficient and represents the tissue drug concentration divided by the plasma drug concentration at steady state. Given the challenges in the experimental measurements of this parameter, several semi-empirical models have been developed over the years, describing $P_{t:p}$ as a function of drug and tissue properties [1]. In this work, the Berezhkovskiy model, given in Equation 2, was used [2]:

$$P_{t:p} = \frac{D_{v,ow} \cdot (V_{nl,t} + 0.3 \cdot V_{ph,t}) + (V_{w,t}/fu_t + 0.7 \cdot V_{ph,t})}{D_{v,ow} \cdot (V_{nl,p} + 0.3 \cdot V_{ph,p}) + (V_{w,p}/fu_p + 0.7 \cdot V_{ph,p})} \tag{2}$$

$V_{nl,t}$ and $V_{nl,p}$ are the volume fractions of neutral lipids in tissue and plasma, respectively; $V_{ph,t}$ and $V_{ph,p}$ are the volume fractions of phospholipids in tissue and plasma; $V_{w,t}$ and $V_{w,p}$ are the water volume fractions in tissue and plasma. Volume fractions are reported in Table 1. $D_{v,ow}$ is the drug partition coefficient between vegetable oil and water and it was obtained as follows $\log D_{v,ow} = 1.115 \cdot \log P_{o,w}$, with $\log P_{o,w}$ the octanol to water partition coefficient [3]. fu_p and fu_t are the drug fraction unbound in plasma and tissue, with the latter calculated as: $fu_t = 1/(1 + 0.5 \cdot (1 - fu_p)/fu_p)$ [3]. All the drug related parameters are given in Table 3.

*darwich@kth.se

The equations for the lungs, arterial and venous blood are reported in equation system 3.

$$
\begin{aligned}
\frac{dx_{lungs}}{dt} &= Q_{tot}\left(\frac{x_{ven}}{V_{ven}} - \frac{x_{lungs}/V_{lungs}}{P_{lungs:p}/B:P}\right)\\
\frac{dx_{art}}{dt} &= Q_{tot}\left(\frac{x_{lungs}/V_{lungs}}{P_{lungs:p}/B:P} - \frac{x_{art}}{V_{art}}\right)\\
\frac{dx_{ven}}{dt} &= \sum_{t\in\mathcal{T}}\left[Q_t\left(\frac{x_t/V_t}{P_{t:p}/B:P}\right)\right] - Q_{tot}\cdot\frac{x_{ven}}{V_{ven}}
\end{aligned}
\tag{3}
$$

Subscript *ven* stands for venous blood. $\mathcal{T}$ represents all the tissues except lungs, arterial and venous blood, small and large intestine, stomach, spleen and pancreas. The difference between lung and Equation 1 is that the lungs receive the input from venous blood with a flux equal to Q_{tot}, or cardiac output. The arterial blood compartment receives its input from the lungs, while the venous blood compartment receives its input from the outputs of all organs defined in $\mathcal{T}$.

Midazolam (MDZ) is primarily metabolised in the liver by the two enzymes, CYP3A4 and CYP3A5. For MDZ both enzymes catalyse two reactions, leading to the formation of two metabolites, *1-hydroxy midazolam* (1-OH-MDZ) and *4-hydroxy midazolam* (4-OH-MDZ) [4, 5]. For this reason, two mass flows corresponding to MDZ metabolism leave the PBPK system from the liver compartment following intravenous drug administration, as represented in Equation system 4.

$$
\begin{aligned}
\frac{dx_{liv}}{dt} &= Q_{liv}\left(\frac{x_{art}}{V_{art}} - \frac{x_{liv}/V_{liv}}{P_{liv:p}/B:P}\right) + \sum_{t\in\mathcal{S}}\left[Q_t\left(\frac{x_t/V_t}{P_{t:p}/B:P}\right)\right]\\
&\quad - MET_{3A4} - MET_{3A5}\\
MET_{3A4} &= \frac{\tilde{V}_{max,3A4,1-OH}\cdot c_{u,liv}}{K_{M,3A4,1-OH}+c_{u,liv}} + \frac{\tilde{V}_{max,3A4,4-OH}\cdot c_{u,liv}}{K_{M,3A4,4-OH}+c_{u,liv}}\\
MET_{3A5} &= \frac{\tilde{V}_{max,3A5,1-OH}\cdot c_{u,liv}}{K_{M,3A5,1-OH}+c_{u,liv}} + \frac{\tilde{V}_{max,3A5,4-OH}\cdot c_{u,liv}}{K_{M,3A5,4-OH}+c_{u,liv}}
\end{aligned}
\tag{4}
$$

Subscript *liv* stands for liver, $\mathcal{S}$ represents the splanchnic organs (spleen, pancreas, stomach, small and large intestine). $c_{u,liv}$ is the unbound liver concentration, equal to $x_{liv}\cdot fu_t/V_{liv}$, where fu_t is the fraction unbound drug in the tissue. MET_{3A4} and MET_{3A5} are the fluxes representing the reactions catalysed by CYP3A4 and CYP3A5. Subscripts $1-OH$ and $4-OH$ refer to the reactions leading to the formation of 1-OH-MDZ and 4-OH-MDZ. All the chemical reactions are described using *Michaelis-Menten* equations [6], where $\tilde{V}_{max}$ is the *in vivo* maximum reaction rate and K_M is the substrate concentration at which the rate is half of $\tilde{V}_{max}$. $\tilde{V}_{max}$ is function of the *in vivo* enzyme abundance and is derived in equation 5, as per [7].

$$
\tilde{V}_{max} = V_{max}\cdot[CYP]\cdot MPPGL\cdot W_{liv} \tag{5}
$$

V_{max} is the experimentally determined *in vitro* maximum rate per amount of CYP isoform, in $(pmol/min)/(pmol\,CYP)$. $[CYP]$ is the enzyme amount per amount of microsomal protein[1] (MP), in $(pmol\,CYP)/(mg\,MP)$. $MPPGL$ is the amount of microsomal protein per gram of liver, in $(mg\,MP)/(g\,liver)$. Finally, W_{liv} is the liver weight in grams.

[1]Vesicles derived from the endoplasmic reticulum abundant in drug metabolising enzymes.

For simulating the pharmacokinetics in a given population of subjects, the PBPK model parameters, such as organ volumes and blood flows, need to be generated reflecting the population distribution. We developed a simple algorithm for generating the organ volumes and blood flows. Briefly:

1. the sex of the subject is extracted;
2. according to the sex, the mean cardiac output and the parameters for height and body mass index (BMI) distributions are fixed;
3. height and BMI of the subject are extracted;
4. the body weight (BW, in kg) is calculated as $BW = BMI \cdot h^2$, where h is the height in m;
5. the cardiac output (CO) is calculated as $CO = \left(\frac{h}{h_{mean}}\right)^{0.75} \cdot CO_{mean}$, with h_{mean} and CO_{mean} the subjects' mean height and cardiac output, respectively [8];
6. organ weights and blood flows were derived by multiplying BW and CO for the respective organs fractions, given in Table 2;
7. organs volumes were derived by dividing the organ weights with organ densities, reported in Table 1.

2 PBPK parameters

Table 1: Organs composition

**Organs**	neutral lipids fraction [3]	phospholipids fraction [3]	water fraction [3]	organ density[c]
Adipose	0.79	0.002	0.18	0.916
Bone	0.074	0.0011	0.439	1.4303
Brain	0.051	0.0565	0.77	1.0365
Heart	0.0115	0.0166	0.758	1.03
Muscle	0.0238	0.0072	0.76	1.041
Skin	0.0284	0.0111	0.718	1.1754
Spleen	0.0201	0.0198	0.788	1.054
Kidney	0.0207	0.0162	0.783	1.05
Gonads[a]	0.0048	0.01	0.8	1[e]
Lung	0.003	0.009	0.811	1.0515
Stomach[b]	0.0487	0.0163	0.718	1.046
Small intestine[b]	0.0487	0.0163	0.718	1.046
Large intestine[b]	0.0487	0.0163	0.718	1.046
Liver	0.0348	0.0252	0.751	1.08[f]
Pancreas	0.0403[d]	0.009[d]	0.641[d]	1.045
Plasma	0.0035	0.00225	0.945	1[e]

[a] Values taken from *Open Systems Pharmacology suite* version 7.1.
[b] Values for stomach, small and large intestine were supposed equal.
[c] Calculated using specific gravity values from [9], considering that water density is 1 kg/L.
[d] values taken from [10, 11]
[e] Gonads and blood density were fixed to 1.
[f] Value taken from [12].

Table 2: Organs weight, blood flows and blood content

**Organs**	weight fraction[b]		blood flow fraction[a]		blood fraction[c]	
	male	female	male	female	male	female
Adipose	0.2040	0.3220	0.0530	0.0900	0.05	0.0850
Bone	0.1620	0.1520	0.0530	0.0500	0.07	0.07
Brain	0.0210	0.0230	0.1280	0.130	0.012	0.012
Heart	0.0057	0.0055	0.0430	0.05	0.01	0.01
Muscle	0.4430	0.3380	0.1810	0.12	0.14	0.105
Skin	0.0520	0.0450	0.0530	0.05	0.03	0.03
Spleen	0.0033	0.0037	0.0320	0.03	0.014	0.0104
Kidney	0.0060	0.0067	0.2170	0.2	0.02	0.02
Gonads	0.0006	0.0002	0.0005	0.0002	0.0004	0.0002
Lung	0.0180	0.0170	1	1	0.1050	0.1050
Stomach	0.0023	0.0027	0.0110	0.01	0.01	0.01
Small intestine	0.0100	0.0120	0.1060	0.12	0.038	0.038
Large intestine	0.0056	0.0069	0.0430	0.05	0.022	0.022
Liver	0.0320	0.0320	0.0690	0.07	0.1	0.1
Pancreas	0.0026	0.0028	0.0110	0.01	0.006	0.006
Blood	0.0767[d]	0.0683[d]	-	-	(0,06,0.18)[e]	(0.06,0.18)[e]

[a] Organ weight fraction (including blood content) on total body weight [8].
[b] Fraction of cardiac output directed to each organ [8].
[c] Fraction of blood weight (relative to total blood weight) [13].
[d] Blood fraction on total body weight [13].
[e] (arterial fraction, venous fraction) [13].

Table 3: Midazolam related parameters

**Parameters**	value	units	references
$B : P$	0.66		[14]
fu_p	0.0303		[14]
molecular weight	325.77	g/mol	[15]
$log_{10} P_{ow}$	3.13		[15]
$V_{max,3A4,1}$	1.96	$pmol/min/(pmol\, CYP)$	[5]
$K_{M,3A4,1}$	2.69	μM	[5]
$V_{max,3A4,4}$	2.52	$pmol/min/(pmol\, CYP)$	[5]
$K_{M,3A4,4}$	29	μM	[5]
$V_{max,3A5,1}$	6.7	$pmol/min/(pmol\, CYP)$	[5]
$K_{M,3A5,1}$	10.7	μM	[5]
$V_{max,3A5,4}$	0.52	$pmol/min/(pmol\, CYP)$	[5]
$K_{M,3A5,4}$	12.1	μM	[5]

3 Convergence of the Kucherenko indices

Figures 1 to 7 detail the convergence of the Kucherenko indices for the various models that were examined.

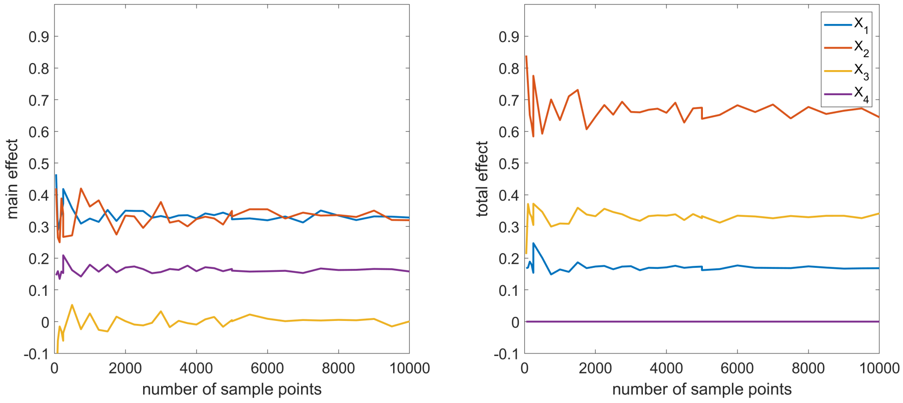

Figure 1: Convergence plot for the Kucherenko indices of the algebraic model 1, with $\rho = 0.7$

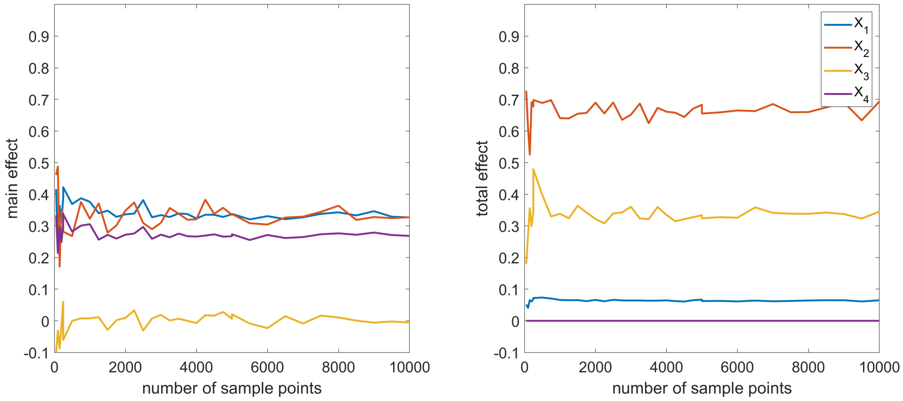

Figure 2: Convergence plot for the Kucherenko indices of the algebraic model 1, with $\rho = 0.9$

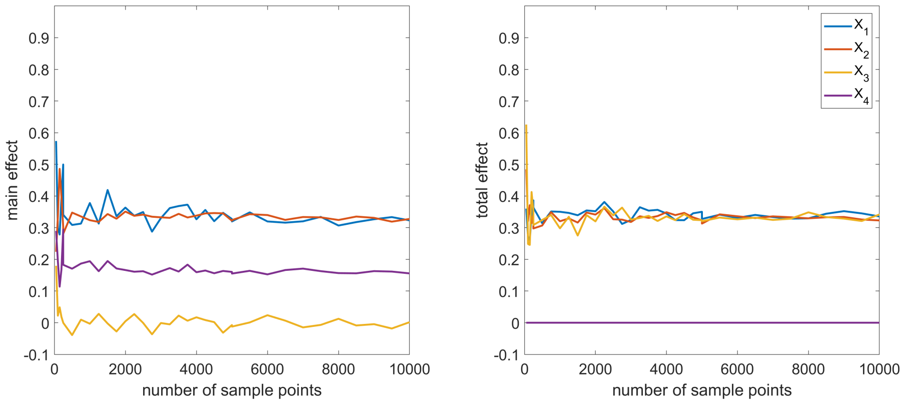

Figure 3: Convergence plot for the Kucherenko indices of the algebraic model 2, with $\rho = 0.7$

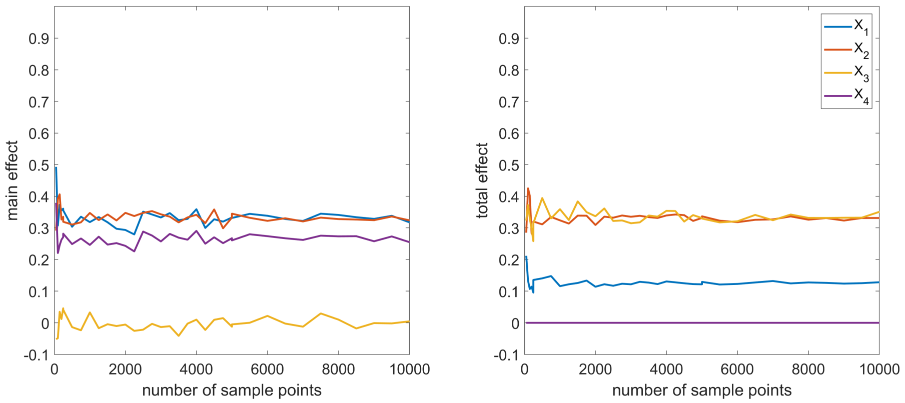

Figure 4: Convergence plot for the Kucherenko indices of the algebraic model 2, with $\rho = 0.9$

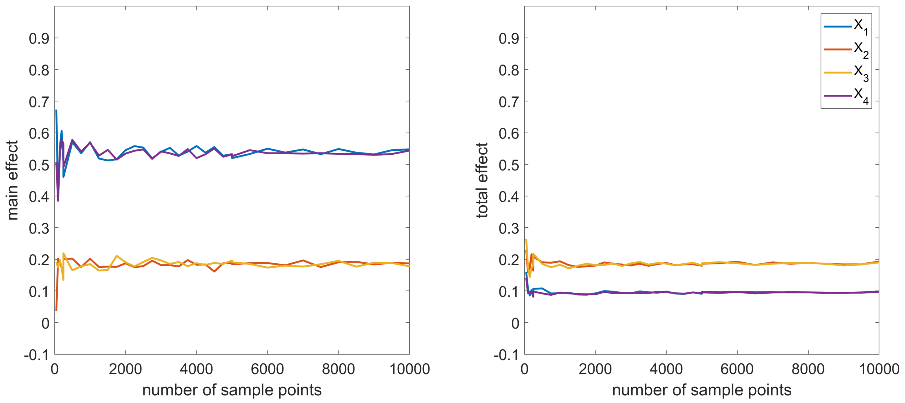

Figure 5: Convergence plot for the Kucherenko indices of the algebraic model 3, with $\rho = 0.7$

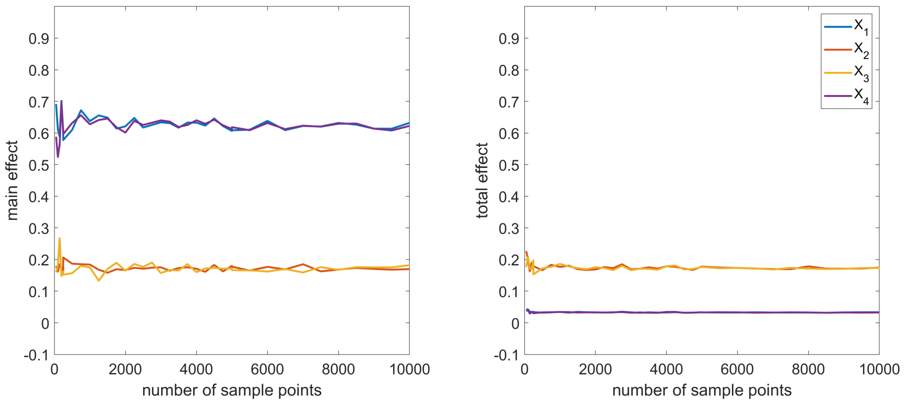

Figure 6: Convergence plot for the Kucherenko indices of the algebraic model 3, with $\rho = 0.9$

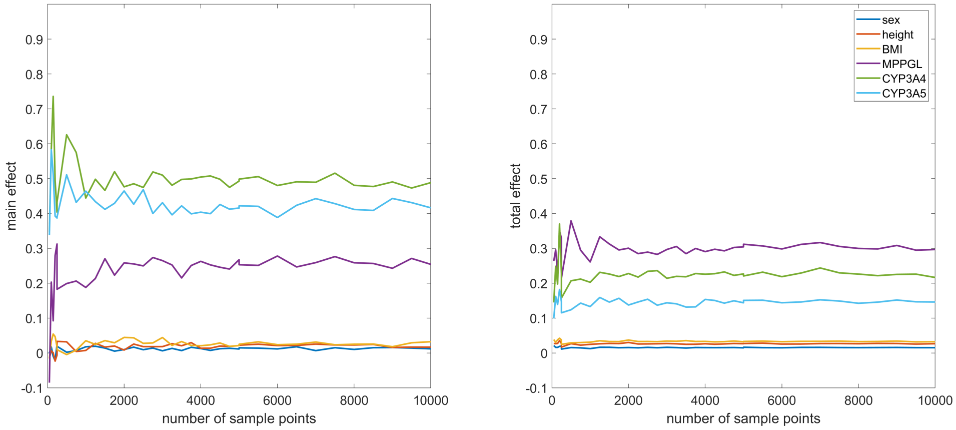

Figure 7: Convergence plot for the Kucherenko indices of the PBPK model for subjects expressing CYP3A5

4 Monte Carlo PBPK model results

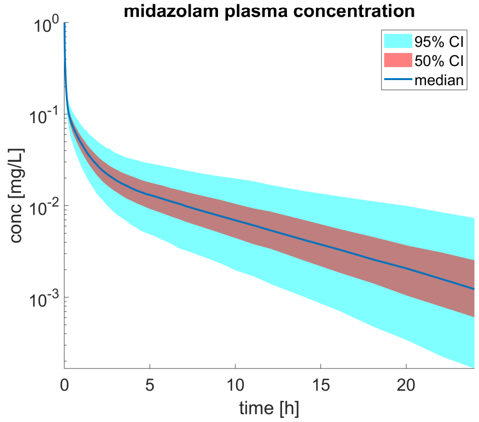

Figure 8: Simulated population midazolam plasma concentration over time following an intravenous (IV) bolus dose of 5 mg. The simulation was performed with the PBPK model for 10,000 individuals. The physiological correlation was considered between the abundances of CYP3A4 and CYP3A5.

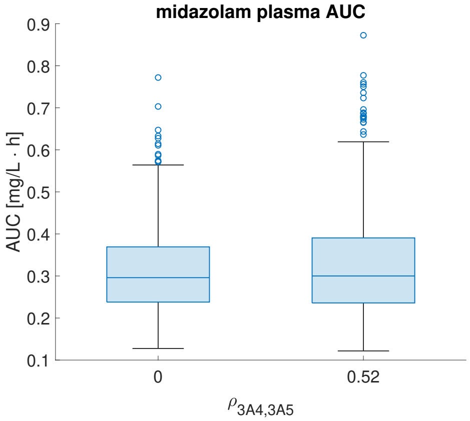

Figure 9: Simulated midazolam AUC distribution both in presence and absence of correlation between CYP3A4 and CYP3A5 abundances. The simulation was performed with the PBPK model for 10,000 individuals.

References

[1] Helen Graham, Mike Walker, Owen Jones, James Yates, Aleksandra Galetin, and Leon Aarons. Comparison of in-vivo and in-silico methods used for prediction of tissue: plasma partition coefficients in rat. *Journal of Pharmacy and Pharmacology*, 64(3):383–396, 2012.

[2] Leonid M. Berezhkovskiy. Volume of Distribution at Steady State for a Linear Pharmacokinetic System with Peripheral Elimination. *Journal of Pharmaceutical Sciences*, 93(6):1628–1640, June 2004.

[3] Patrick Poulin and Frank-Peter Theil. Prediction of pharmacokinetics prior to in vivo studies. 1. Mechanism-based prediction of volume of distribution. *Journal of Pharmaceutical Sciences*, 91(1):129–156, January 2002.

[4] Michaela Vossen, Michael Sevestre, Christoph Niederalt, In-Jin Jang, Stefan Willmann, and Andrea N. Edginton. Dynamically simulating the interaction of midazolam and the CYP3A4 inhibitor itraconazole using individual coupled whole-body physiologically-based pharmacokinetic (WB-PBPK) models. *Theoretical Biology and Medical Modelling*, 4(1):13, March 2007.

[5] Aleksandra Galetin, Caroline Brown, David Hallifax, Kiyomi Ito, and J. Brian Houston. Utility of recombinant enzyme kinetics in prediction of human clearance: Impact of variability, cyp3a5, and cyp2c19 on cyp3a4 probe substrates. *Drug Metabolism and Disposition*, 32(12):1411–1420, 2004.

[6] Michaelis L. and Menten M.L. Die Kinetik der Invertinwirkung. *Biochemistry Zeitung*, 49:333 – 369, 1913.

[7] Amin Rostami-Hodjegan and Geoffrey T. Tucker. Simulation and prediction of in vivo drug metabolism in human populations from in vitro data. *Nature Reviews Drug Discovery*, 6(2):140–148, February 2007. Number: 2 Publisher: Nature Publishing Group.

[8] Stefan Willmann, Karsten Höhn, Andrea Edginton, Michael Sevestre, Juri Solodenko, Wolfgang Weiss, Jörg Lippert, and Walter Schmitt. Development of a Physiology-Based Whole-Body Population Model for Assessing the Influence of Individual Variability on the Pharmacokinetics of Drugs. *Journal of Pharmacokinetics and Pharmacodynamics*, 34(3):401–431, June 2007.

[9] Ronald P. Brown, Michael D. Delp, Stan L. Lindstedt, Lorenz R. Rhomberg, and Robert P. Beliles. Physiological Parameter Values for Physiologically Based Pharmacokinetic Models. *Toxicology and Industrial Health*, 13(4):407–484, July 1997.

[10] Trudy Rodgers, David Leahy, and Malcolm Rowland. Physiologically Based Pharmacokinetic Modeling 1: Predicting the Tissue Distribution of Moderate-to-Strong Bases. *Journal of Pharmaceutical Sciences*, 94(6):1259–1276, June 2005.

[11] Trudy Rodgers and Malcolm Rowland. Physiologically based pharmacokinetic modelling 2: Predicting the tissue distribution of acids, very weak bases, neutrals and zwitterions. *Journal of Pharmaceutical Sciences*, 95(6):1238–1257, June 2006.

[12] Axel Heinemann, Friedel Wischhusen, Klaus Püschel, and Xavier Rogiers. Standard liver volume in the caucasian population. *Liver Transplantation and Surgery*, 5(5):366–368, 1999.

[13] J. Valetin. Basic Anatomical and Physiological Data for Use in Radiological Protection: Reference Values. Technical Report 89, International Commission on Radiological Protection (ICRP), 2002.

[14] MJE Brill, PAJ Välitalo, AS Darwich, B van Ramshorst, HPA van Dongen, A Rostami–Hodjegan, M Danhof, and CAJ Knibbe. Semiphysiologically based pharmacokinetic model for midazolam and cyp3a mediated metabolite 1-oh-midazolam in morbidly obese and weight loss surgery patients. *CPT: Pharmacometrics & Systems Pharmacology*, 5(1):20–30, 2016.

[15] Open Systems Pharmacology (OSP). OSP Suite - Version 7.1, (http://www.open-systems-pharmacology.org/), 2017.
